# XLS (c9orf142) is a new component of mammalian DNA double-stranded break repair

**DOI:** 10.1038/cdd.2015.22

**Published:** 2015-03-13

**Authors:** A Craxton, J Somers, D Munnur, R Jukes-Jones, K Cain, M Malewicz

**Affiliations:** 1 MRC Toxicology Unit, Hodgkin Building, Lancaster Road, Leicester LE1 9HN, UK

## Abstract

Repair of double-stranded DNA breaks (DSBs) in mammalian cells primarily occurs by the non-homologous end-joining (NHEJ) pathway, which requires seven core proteins (Ku70/Ku86, DNA-PKcs (DNA-dependent protein kinase catalytic subunit), Artemis, XRCC4-like factor (XLF), XRCC4 and DNA ligase IV). Here we show using combined affinity purification and mass spectrometry that DNA-PKcs co-purifies with all known core NHEJ factors. Furthermore, we have identified a novel evolutionary conserved protein associated with DNA-PKcs—c9orf142. Computer-based modelling of c9orf142 predicted a structure very similar to XRCC4, hence we have named c9orf142—XLS (XRCC4-like small protein). Depletion of c9orf142/XLS in cells impaired DSB repair consistent with a defect in NHEJ. Furthermore, c9orf142/XLS interacted with other core NHEJ factors. These results demonstrate the existence of a new component of the NHEJ DNA repair pathway in mammalian cells.

Double-stranded DNA breaks (DSBs) are among the most cytotoxic DNA lesions for mammalian cells.^1^ Effective repair of DSBs is essential for cellular survival and for suppression of potential deleterious chromosomal rearrangements.^2^ Two main DNA repair pathways eliminate DSBs—homologous recombination (HR) or non-homologous end joining (NHEJ). HR utilises an undamaged copy of the chromosome as a template to direct repair, thus this restricts HR to the S and G2/M phases of the cell cycle, when such an extra chromosome copy is available.^3^ NHEJ performs the bulk of DSB repair in mammalian cells and in particular in during the G1 phase of the cell cycle, where the cells are completely dependent on NHEJ. NHEJ can be further subdivided into so-called classical NHEJ (c-NHEJ) and alternative NHEJ (alt-NHEJ).^4^ These DNA repair pathways utilise distinct protein components and also show different efficiencies of end ligation. In general, c-NHEJ is much more effective in end ligation than alt-NHEJ and can ligate most unrelated DNA ends directly or with minimal processing. In contrast alt-NHEJ requires short microhomologies between the DNA ends for ligation.^5^ C-NHEJ requires the following seven core proteins: Ku70/Ku86 dimers, DNA-PKcs (DNA-dependent protein kinase catalytic subunit), Artemis nuclease, XRCC4-like factor (XLF) and the XRCC4/ligase IV complex.^6, 7^ The DSB repair during c-NHEJ is initiated by the Ku dimer that senses the presence of free double-stranded DNA ends in cells and rapidly binds such ends with high affinity. DNA-bound Ku then recruits DNA-PKcs (DNA-PKcs/Ku70/Ku86 complex is termed DNA-PK holoenzyme), which has a protein kinase activity and is required for activation of the nuclease Artemis.^8^ Artemis, in turn, is responsible for DNA end processing in order to achieve DNA end structures suitable for ligation. The final step of c-NHEJ is the ligation of processed DNA ends by XRCC4/ligase IV complex. This final step is stimulated by XLF protein that interacts with XRCC4 forming long filamentous structures at DSBs to facilitate DNA end joining.^9, 10^ XRCC4 and XLF factors are distinct among NHEJ factors in that they share similar tertiary structure but show low primary sequence conservation.^11^ Since the identification of XLF in 2006, no new core factors have been discovered.^11, 12^ Importantly, c-NHEJ is essential for proper development, as mutations in this pathway lead to immunodeficiency and defective neurogenesis in humans.^7^ It is therefore essential to fully decipher the identity of components for the c-NHEJ pathway and their regulation.

In this study, proteomic analysis of DNA-PKcs-containing protein complexes identified an abundant previously uncharacterised protein c9orf142, which we have named c9orf142—XLS (XRCC4-like small protein). Structural modelling predicts XLS to be highly similar to XRCC4 and XLF, and depletion of XLS delays ionising radiation (IR)-induced DNA DSB repair. Moreover, XLS is associated with other core c-NHEJ factors. Our data strongly suggest that c9orf142/XLS represents a novel c-NHEJ component in mammalian cells.

## Results

**Figure 1:**
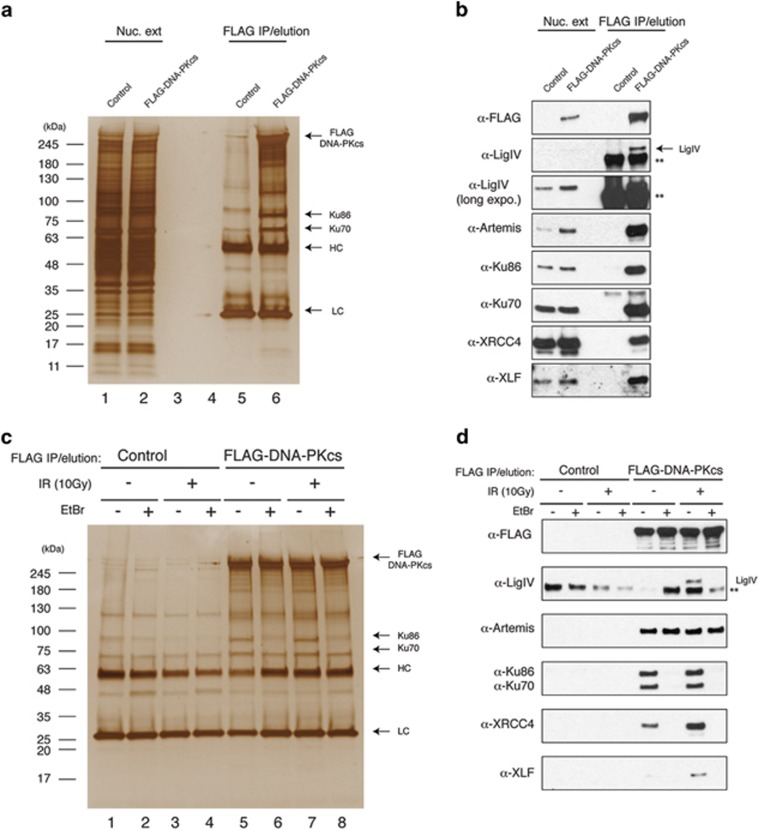
DNA-PKcs co-purifies with all known core NHEJ components. (**a**) Nuclear extracts (lanes 1 and 2) or eluates of purified anti-FLAG IPs from either HEK293 control (lane 5) or FLAG-DNA-PKcs-expressing cells (lane 6) were resolved by SDS-PAGE and visualised by silver staining. (**b**) As described in **a**, except that core NHEJ proteins were detected by immunoblotting. **Position of unspecific protein band. (**c**) Eluates of purified anti-FLAG IPs from either untreated (lanes 1 and 2) or irradiated (10 Gy, lanes 3 and 4) HEK293 control, or untreated (lanes 5 and 6) or irradiated (10 Gy, lanes 7 and 8) FLAG-DNA-PKcs-expressing cells were resolved by SDS-PAGE and visualised by silver staining. Nuclear extracts were incubated in the absence (lanes 1, 3, 5 and 7) or presence (lanes 2, 4, 6 or 8) of EtBr (0.2 mg/ml) for 30 min before addition of anti-FLAG M2 beads. (**d**) As described in **c**, except that core NHEJ proteins were detected by immunoblotting. **Position of unspecific protein band

**Figure 2:**
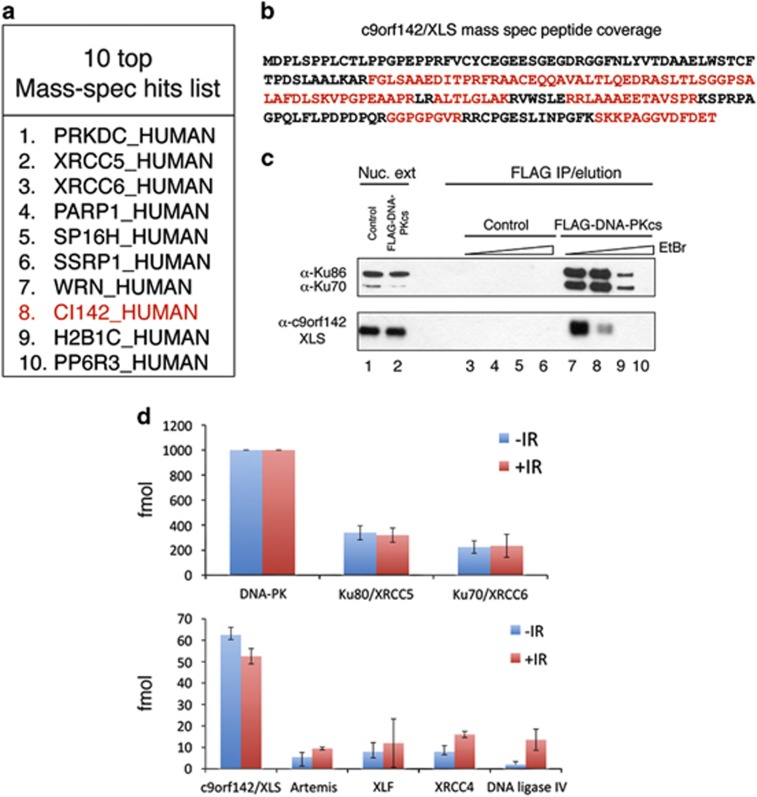
An uncharacterised protein c9orf142 associates with DNA-PKcs-containing NHEJ complexes. (**a**) Table showing 10 most abundant proteins identified in anti-FLAG IPs from FLAG-DNA-PKcs but not HEK293 control cells (average protein abundance from two independent experiments was used in creation of the ranking list). C9orf142 is shown in red (proteins are named based upon UniProt nomenclature. CI142_HUMAN indicates c9orf142/XLS). (**b**) Sequence coverage for c9orf142/XLS. Amino acids, which were identified within peptides detected by LC-MS/MS, are marked in red. The total % sequence coverage was 49%. (**c**) Nuclear extracts (lanes 1 and 2) or eluates of isolated anti-FLAG IPs from nuclear extracts from either HEK293 control (lanes 3–6) or FLAG-DNA-PKcs expressing cells (lanes 7–10), which had been incubated with increasing concentrations of EtBr before addition of anti-FLAG beads were resolved by SDS-PAGE. Ku70, Ku86 and c9orf142 were detected by immunoblotting. (**d**) Identification of core NHEJ proteins from FLAG-DNA-PKcs IPs by LC-MS/MS mass spectrometry. Results shown in each column are the average quantities (fmol) of the indicated protein detected from duplicate injections from a total of four independent experiments

To identify and characterise novel DNA-PKcs-interacting proteins, we have generated a HEK293 cell line stably expressing N-terminal FLAG-tagged DNA-PKcs at physiological expression levels. Diluted nuclear extracts from these cells were subject to anti-FLAG immunoprecipitation (IP) followed by FLAG peptide elution and analysis by SDS-PAGE and silver staining (Figure 1a). FLAG-DNA-PKcs IPs contained several visible protein bands compared with control samples including 70 and 85 kDa species, presumably corresponding to Ku70/Ku86 subunits of the DNA-PK holoenzyme. Immunoblotting of eluates from control and FLAG-DNA-PKcs IPs identified all known core NHEJ components (Ku70/Ku86, Artemis, DNA Ligase IV, XRCC4 and XLF) specifically in FLAG-DNA-PKcs IPs (Figure 1b). Cellular DNA damage induced by IR is known to affect the stability of various DNA repair complexes by eliciting activation of stress signalling pathways, which catalyse many protein post-translational modifications.^3^ Therefore, further experiments were performed under basal conditions or after exposing samples to IR. In order to assess the effect that contaminating DNA had on complex assembly, additional samples were treated with ethidium bromide (EtBr) before elution (Figure 1c). Although IR did not show obvious visible changes to the distribution of silver-stained bands, EtBr led to disappearance of several prominent protein bands including the 70- and 85-kDa species, which we believe represent both Ku proteins (Figure 1c). Western blotting of core NHEJ factors revealed that Artemis directly interacted with DNA-PKcs as shown previously.^13^ All other core NHEJ proteins interacted with DNA-PKcs in a DNA-dependent fashion (Figure 1d). Interestingly, IR significantly increased the abundance of XLF and XRCC4/DNA ligase IV factors associated with DNA-PKcs, whereas the levels of Ku70/Ku86 and Artemis remained largely unchanged (Figure 1d). We reasoned that based upon our ability to detect all known core NHEJ factors in the FLAG-DNA-PKcs IPs, it is possible that we may also detect previously uncharacterised core NHEJ components. To systematically analyse the protein composition of FLAG-DNA-PKcs IPs, eluates were analysed by mass spectrometry to determine their proteomic composition. This proteomic analysis revealed the presence of large amounts of Ku proteins associated with DNA-PKcs (Figures 2a and d). The other most abundant proteins were PARP1, the FACT complex (SP16H and SSRP1) Werner protein (WRN) and protein phosphatase 6 regulatory subunit 3 (PP6R3) as previously shown (Figure 2a).^14^ Importantly, we also found substantial quantities of a previously uncharacterised protein c9orf142 consistently co-purifying with FLAG-DNA-PKcs (Figures 2a, b and d), which we have named XLS. Notably, compared with other core NHEJ factors, XLS was greater than fourfold, more abundant than XRCC4, XLF, DNA ligase IV and Artemis in the FLAG-DNA-PKcs IPs, and its levels were not increased in response to radiation in contrast to XRCC4, XLF and DNA ligase IV (Figures 1d and 2d). The presence of XLS in DNA-PKcs IPs was verified by western blotting (Figure 2c). These results also demonstrated that XLS associated with DNA-PKcs in a DNA-dependent manner, although interestingly the concentration of EtBr required for stripping XLS from the beads was significantly lower than the amount of EtBr needed for complete removal of Ku factors (Figure 2c).

**Figure 3:**
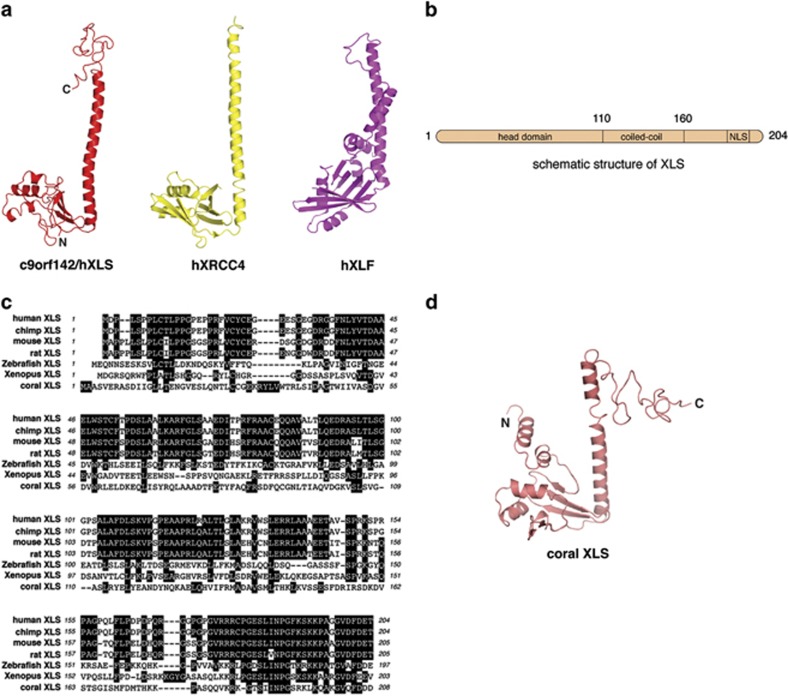
Computer modelling of c9orf142/XLS protein predicts a structure similar to XRCC4. (**a**) 3D structural model for full-length human c9orf142/XLS generated using the Phyre2 server, which predicted with 95% and 85% confidence overlapping structural features between amino acids 5–160 of human c9orf142/XLS with human XRCC4 and XLF (crystal structures), respectively. Although XRCC4 and XLF typically form dimers, the figure shows monomers for all proteins to highlight their structural similarities. ‘N' and ‘C' indicate positions of the N- and C terminus of XLS, respectively. (**b**) Schematic figure showing the predicted structural and function domains for c9orf142/XLS. These are a N-terminal head domain, a middle coiled-coil domain and a C-terminal nuclear localisation signal (NLS). (**c**) Primary sequence alignment of c9orf142/XLS from human, chimpanzee, mouse, rat, zebrafish, *Xenopus* and starlet sea anemone (coral) species. Identical amino acids are indicated as shaded black areas. (**d**) Predicted 3D structural model generated using Phrye2 server for full-length starlet sea anemone c9orf142/XLS, which shares ~20% sequence identity with human XLS (not shown)

We were intrigued by the high relative abundance of XLS in our DNA-PKcs IPs. Although primary sequence analysis of XLS did not reveal significant similarities to any known proteins, computer-based structural modelling using the Phyre2 algorithm^15^ revealed a structure of high similarity to XRCC4 (Figure 3a). It also showed structural resemblance to XLF, a known XRCC4 interactor, that forms filament-like structures with XRCC4.^9, 10^ Although XLS is significantly smaller that XRCC4 (204 aa compared with 334 aa), it maintains similar overall predicted domain structure characterised by the presence of a N-terminal head domain followed by a centrally located extended coiled coil and a C-terminal predicted bipartite nuclear localisation sequence (Figure 3b). The Phyre2 algorithm modelled amino acids 8–160 of XLS (ca. 75% of the full-length XLS) with high confidence (ca. 95%). However, the C-terminal portion of XLS (aa 161−204) could not be modelled with high confidence and hence remained unstructured (Figure 3a). Modelling results obtained with the use of Phyre2 server were confirmed by subjecting XLS sequences to I-TASSER computer modelling platform (data not shown). The primary sequence of XLS is strongly phylogenetically conserved (Figure 3c) with surprisingly high-sequence conservation in the C-terminal region of XLS, which might suggest essential functional and/or regulatory roles for this region of the protein. Evolutionary conservation of XLS structure can be further exemplified by its comparison with coral XLS orthologue. Although conservation of the protein sequence between the human and coral XLS is low (ca. 20% Figure 3c), the model for the coral XLS orthologue generated using the Phyre2 algorithm also predicted with high confidence similarity to XRCC4 (ca. 95% model confidence; Figure 3d).

**Figure 4:**
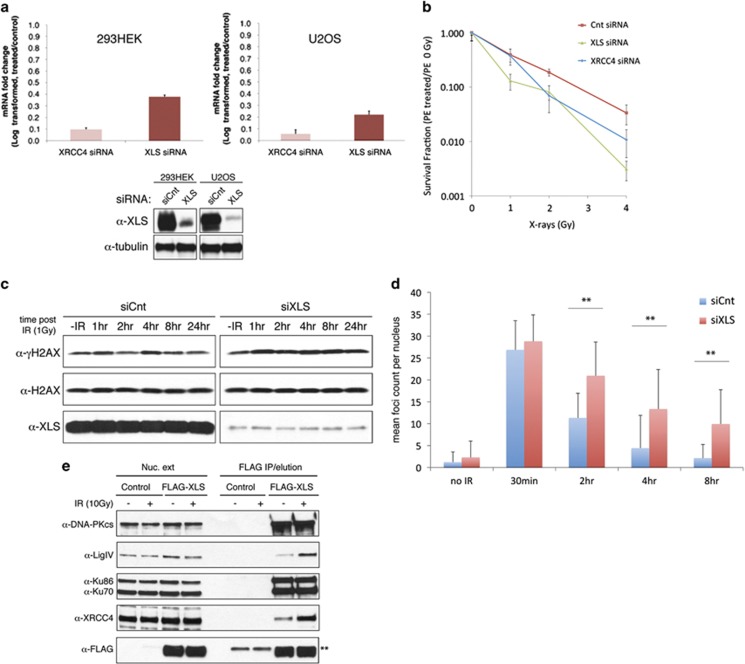
c9orf142/XLS protein interacts with most core NHEJ factors and its depletion leads to defective DNA DSB repair. (**a**) Upper panels: qPCR of XRCC4 and XLS mRNA expression after 72 h siRNA transfection in 293H and U2OS cells. Samples were normalised for GAPDH mRNA expression and expressed as mRNA fold change relative to control siRNA samples. Mean values and S.D. were plotted from three experimental repeats. Lower panels: western blot for XLS in control and XLS siRNA-transfected HEK293H and U2OS cells. (**b**) Clonogenic survival assay of 293H cells transfected with XLS, XRCC4 or control (Cnt) siRNA. Survival fraction was determined relative to 0 Gy control samples. Experiment was repeated three times. Representative experiment is shown. Mean values and S.D. were plotted. (**c**) XLS knockdown cells have elevated *γ*H2AX levels following IR. U2OS cells were irradiated with X-rays (1 Gy) or left untreated. After the indicated times, histones were extracted from cells and immunoblotted for *γ*H2AX or H2AX (loading control). XLS immunoblots were performed using the Triton X-100-soluble fractions. (**d**) XLS knockdown cells show slow *γ*H2AX foci resolution following IR. U2OS cells were irradiated with X-rays (1 Gy) or left untreated (no IR). *γ*H2AX foci were visualised by immunofluorescence. Experiment was repeated four times. Representative experiment is shown. Mean foci values per nucleus and S.D. were plotted. **P<0.005. (**e**) XLS interacts with core c-NHEJ factors. HEK293S cells were transiently transfected for 20 h with either pCMX-LacZ (control) or pCMX-FLAG-XLS. Cells were untreated or irradiated with 10 Gy X-ray. After 30 min recovery at 37 °C, nuclear extracts were isolated. Following anti-FLAG IP and FLAG peptide elution, FLAG-XLS and associated proteins were eluted, resolved by SDS-PAGE and immunoblotted for the indicated core c-NHEJ factors. **Position of IgG light chains cross-reacting with the secondary antibodies

The high relative abundance of XLS in DNA-PKcs IPs and its predicted striking structural similarity to XRCC4 led us to hypothesise that XLS may be involved in DSB repair. In order to assess the function of XLS, we first depleted XLS (and XRCC4 as a reference) using siRNA in two human cell types and observed efficient knockdown of XLS in both cell lines by qPCR and immunoblotting (Figure 4a). A typical phenotype of the NHEJ pathway deficiency is cellular radiosensitivity.^7^ Notably, knockdown of XLS in cells produced a radiosensitivity phenotype similar to depletion of XRCC4 measured by clonogenic survival assays (Figure 4b). To further establish similarity between XLS function and the activity of core NHEJ factors, we took advantage of the observation that XLF-deficient cells show substantially delayed resolution of histone H2AX phosphorylation after radiation.^11^ We therefore assessed the kinetics of histone H2AX phosphorylation after IR in XLS-depleted cells. Knockdown of XLS delayed the disappearance of histone H2AX phosphorylation after IR consistent with defective DNA repair (Figure 4c). In order to confirm the DSB repair defect, we directly scored the repair kinetics of DSBs in XLS-depleted cells by visualisation of nuclear foci stained for phospho-Ser139-H2AX mark, a known DSB marker in eukaryotic cells.^16^ Depleting XLS led to a strong reduction in overall efficiency of DSB repair in cells (Figure 4d), closely resembling the previously reported phenotype of a human XLF mutant cell line.^11^ Collectively, these deficiencies in DSB repair suggest a defect in NHEJ.

To gain further evidence for XLS being a component of NHEJ, FLAG-tagged XLS was immunoprecipitated from nuclear extracts of HEK293S cells and IPs were probed for the presence of core NHEJ factors. Importantly, although XLS co-purified with DNA-PK subunits, XRCC4 and DNA ligase IV, treatment of cells with IR led to substantial increases in XRCC4/Ligase IV in FLAG-XLS IPs (Figure 4e).

## Discussion

Historically most c-NHEJ factors have been discovered through complementation experiments, in which previously isolated mutant cell lines served as a critical starting point.^17^ A notable exception was the discovery of DNA ligase IV, which was found as a XRCC4-associated protein^18^ and the discovery of XLF by the Jackson group^11^ through a two-hybrid approach. Here we have shown that immunoaffinity purification coupled with proteomics analysis by mass spectrometry can be successfully applied to identify new c-NHEJ proteins using a known NHEJ factor as bait (DNA-PKcs in this instance). However, this method is not without caveats. For example, although we could detect Artemis nuclease in our FLAG-DNA-PKcs IPs, the overall yield of Artemis was comparatively low, thereby making it technically challenging to discover Artemis in an unbiased fashion among other proteins of low abundance present in the complex. This is in striking contrast to our discovery of c9orf142/XLS that robustly co-purified with DNA-PKcs and was consistently observed among the most abundant proteins detected (Figures 2a and d).

Here we have demonstrated that c9orf142/XLS is a new factor required for DSB repair in mammalian cells. Besides core proteins, the c-NHEJ DNA repair pathway uses additional so-called accessory factors (e.g., APLF (aprataxin and polynucleotide kinase/phosphatase-like factor) protein and NR4A orphan receptors^19, 20^). These proteins typically serve to increase the efficiency of c-NHEJ without being absolutely essential. It appears possible that XLS represents a genuine core factor for c-NHEJ rather than an accessory molecule. This hypothesis is based on the observation that XLS is structurally similar to both XRCC4 and XLF (Figure 3a). Notably depletion of XLS produces a strong DSB repair defect and increased radiosensitivity, features uncommon for accessory NHEJ factors.^19^ However, more definitive proof for XLS being considered a core factor for c-NHEJ is nevertheless required. Other hallmarks of c-NHEJ deficiency include a shift in DSB repair to microhomology^21^ and a defect in somatic VDJ recombination in lymphocytes.^22^ With regard to VDJ recombination, it will be essential to assess the efficiency of this process in XLS knockout mice or potential human patients bearing inactivating mutations in the XLS-encoding gene. Precisely, how XLS functions within the c-NHEJ complex remains to be established. Understanding the pattern of XLS interactions among the c-NHEJ components will be important in deciphering its function in DSB repair. Accordingly, it is intriguing to note that, based on the relative abundance of c-NHEJ factors in our DNA-PKcs IPs, it is interesting to speculate whether XLS may be directly bound to DNA-PKcs or via Ku, as only Ku factors are present in large enough quantities to mediate the bridging of XLS to DNA-PKcs. Given that interaction of XLS with DNA-PKcs is DNA dependent (Figure 2c), it is more likely that XLS binds Ku subunits and/or DNA directly. Interestingly, FLAG-XLS co-purified with other c-NHEJ core factors, consistent with XLS being a core NHEJ component. Clearly, more experimental work needs to be done using XLS-deficient cells to draw a definitive conclusions regarding exact contribution of this protein to mammalian DSB repair.

While this manuscript was under preparation, another group reported the discovery of c9orf142 as a novel c-NHEJ factor. Although their functional data largely overlap with ours, their discovery of c9orf142 occurred through application of bioinformatics- and structure-guided approaches.^23^

## Materials and Methods

### Cells

HEK293 and U2OS cells were cultured in Dulbecco's-modified Eagle's medium, containing 4.5 g/l D-glucose and GlutaMAX (Life Technologies, Carlsbad, CA, USA), and supplemented with 10% fetal bovine serum. HEK293 cell culture medium also contained pyruvate. A FLAG-DNA-PKcs-expressing stable cell line was generated by transfecting a mammalian expression vector encoding FLAG-DNA-PKcs fusion protein (gift from Dr. David J. Chen, UTSouthwestern, Dallas, TX, USA) with pTKHyg plasmid into 293H cells (Life Technologies) and selecting individual clones with 200 *μ*g/ml of hygromycin B (hygB). Individual hygB-resistant clones were screened for full-length FLAG-DNA-PKcs expression by western blotting. One clone expressing physiological levels of tagged-DNA-PKcs was selected for further experiments. Suspension-adapted HEK293 (HEK293S) cells were cultured in Freestyle 293 medium (Life Technologies) supplemented with hygB (100 *μ*g/ml) between densities of 0.5–3 × 10^6^ cells per ml in conical flasks on a shaking platform (160 r.p.m.) in a humidified 37 °C incubator. HEK293S cells were transfected with pCMX-FLAG-XLS or pCMX-LacZ plasmid (gift from Dr. Thomas Perlmann, LICR, Stockholm, Sweden) using Freestyle MAX (Life Technologies) according to the manufacturer's instructions.

### Plasmid constructs

Human full-length c9orf142/XLS cDNA was amplified with Q5 polymerase (NEB, Ipswich, MA, USA) by using oligo-dT-primed cDNA template from U2OS cells. The PCR product was subsequently subcloned into pCMX expression vector (gift from Dr. Thomas Perlmann, LICR). Sanger sequencing confirmed the insert sequence.

### Antibodies

Mouse anti-FLAG (M2) and anti-tubulin (Sigma-Aldrich, St. Louis, MO, USA); mouse anti-Ku70, Ku86, DNA ligase IV, XLF and SPT16H (Santa Cruz Biotechnology, Dallas, TX, USA); rabbit anti-Artemis, PARP1 and *γ*H2AX (Cell Signalling Technology, Danvers, MA, USA); c9orf142/XLS, DNA-PKcs and H2AX (Abcam, Cambridge, UK); and rabbit anti-XRCC4 was a generous gift from Dr. Dik Van Gent (Erasmus University, Rotterdam, Holland).

### Immunoaffinity purification of FLAG-tagged DNA-PKcs and associated proteins from nuclear extracts

For each sample, 200–250 × 10^6^ HEK293S cells stably expressing FLAG-DNA-PKcs or empty vector control were pelleted by centrifugation at 200 × *g*, resuspended in 20 ml fresh media and placed in cell culture dishes and exposed to X-rays at 10 Gy (R320 Cabinet, X-strahl, Surrey, UK) or left untreated. Cells were subsequently incubated for 30 min at 37 °C. All subsequent procedures were performed on ice or at 4 °C. Cells were scraped into 15 ml tubes, centrifuged at 200 × *g* for 5 min and washed twice in ice-cold PBS-MC (PBS, 1 mM MgCl_2_ and 1 mM CaCl_2_). Cells were gently resuspended in 3.6 ml ice-cold hypotonic buffer (10 mM HEPES, pH 7.9, 10 mM KCl, 0.1 mM EDTA and 0.1 mM EGTA supplemented with complete Mini protease inhibitor mixture tablets (Roche Diagnostics, Burgess Hill, UK), 10 mM NaF, 1 mM Na_3_VO_4_, 10 *μ*M MG132, 1 mM DTT and 1 mM phenylmethanesulphonyl fluoride (PMSF)). After incubation for 15 min, cells were vortexed for 10 s, and immediately centrifuged at 2300 × *g* for 5 min at 4 °C. Nuclei were washed with 1 ml hypotonic buffer and re-centrifuged as described above. Pellets were overlayed with 4 ml high salt buffer (20 mM HEPES, pH 7.9, 420 mM NaCl, 1.5 mM MgCl_2_ and 20% glycerol supplemented with complete Mini protease inhibitor mixture tablets (Roche Diagnostics), 10 mM NaF, 1 mM Na_3_VO_4_, 10 *μ*M MG132, 1 mM DTT and 1 mM PMSF) for 15 min with occasional mixing to resuspend crude nuclei. Following centrifugation at 15 000 × *g* for 30 min, high salt nuclear extracts were diluted with 2 volumes of 20 mM HEPES, pH 7.9, 20% glycerol and supplemented with 0.5% Igepal CA630, and subsequently incubated for an additional 30 min and re-centrifuged at 15 000 × *g* for 30 min. Protein concentrations were quantified using Bradford Reagent (Bio-Rad, Hercules, CA, USA) and equal quantities were incubated overnight by end-to-end mixing at 4 °C with 25 *μ*l of low pH glycine-washed packed anti-FLAG M2 agarose beads according to the manufacturer's instructions (Sigma-Aldrich). Beads were washed five times with 20 mM HEPES, pH 7.9, 140 mM NaCl, 0.5 mM MgCl_2_, 20% glycerol, 10 mM NaF, 1 mM Na_3_VO_4_, 10 *μ*M MG132, 1 mM DTT and 1 mM PMSF, and proteins were eluted with 50 *μ*l 3X FLAG peptide (0.2 mg/ml). In some experiments, EtBr was added to either incrementally reduce DNA binding (5–200 *μ*g/ml) or completely suppress DNA-dependent binding (200 *μ*g/ml). Eluates were resolved by SDS-PAGE and gels were visualised with either silver stain (Pierce, Rockford, IL, USA) or for mass spectrometry stained with colloidal Coomassie (National Diagnostics, Hessle, UK).

### Identification of DNA-PKcs-interacting proteins using LC-MS/MS

After destaining with deionised H_2_O according to the manufacturer's instructions, gels were serially sectioned, digested with trypsin and peptides were extracted as described previously.^24, 25^ Dried tryptic peptides were resuspended in 5% formic acid and 10% acetonitrile (9 : 1), spiked with 20 fmol/*μ*l MassPREP standards (Waters Corporation, Manchester, UK), using yeast ADH1 (accession no. P00330) and bovine serum albumin (accession no. P02769), Nanoscale UPLC separation of tryptic peptides was carried out on a nanoAcquity UPLC system (Waters Corporation) equipped with a 25 cm × 75 *μ*m I.D., 1.7 *μ*m, BEH130 C18 analytical reverse phase column. Samples (2–4 *μ*l injections) were separated using 90-min, 3−40% acetonitrile gradients at 0.3 *μ*l/min. Mass spectrometric analysis of eluted peptides, using a Waters Synapt G2-S HDMS mass spectrometer (Waters Ltd, Elstree, UK), equipped with T-Wave-IMS and carried out in data-independent acquisition and ion mobility modes (HDMS^E^), with a travelling wave velocity of 650 m/s. Peptide fragmentation was performed, by stepping between 4 eV (low energy) and 20–50 eV (collision-induced dissociation) voltages. Low-energy and CID data were acquired with a 1-s cycle scan time and 50–2000 *m/z* mass range. LC-MS data were processed and searched using Waters ProteinLynx Global SERVER version 3.0 (PLGS, Waters) and identified, using the UniProt Human reviewed database (UniProtKB release 2014_11, 20265 entries). Raw data files were analysed using PLGS version 3 and ISOQuant.^26^ These data were used for ‘top 3' absolute quantification of proteins.^27^ For database searching in PLGS, peptide mass tolerance and fragment mass tolerance were set to auto, with one missed cleavage and variable modification for methionine oxidation. A false-discovery rate (FDR) of 1% was used for PLGS and for ISOQuant analysis an FDR of 0.1% was used with only TOP 3 peptide hits used for quantification. Data were also analysed using Scaffold version 3.3.1 software (Proteome Software Inc., Portland, OR, USA) as previously described.^25^

### Computer modelling, visualisation and multiple sequence alignment of proteins

Computer modelling was performed by submitting XLS protein sequences to Phyre2 server.^15^ For both human (accession no. NP_899064) and coral (accession no. XP_001641097, starlet sea anemone) XLS models with a high degree of probability of correctness were achieved (ca. 95%). Results obtained with the use of Phre2 server were confirmed by subjecting XLS sequences to I-TASSER computer modelling platform (http://zhanglab.ccmb.med.umich.edu). Figures 3a and d were generated using PyMOL Molecular Graphic System, version 1.6.0.0 (Schrödinger, LLC, Camberley, UK). The Phyre2-generated models of human and coral XLS were compared with the crystal structures of XRCC4; PDB accession codes 1FU1^28^ and XLF; PDB accession code 2R9A.^29^ XLS protein sequences were aligned using ClustalW algorithm from within MacVector sequence analysis software (MacVector Inc., Cary, NC, USA). Human XLS (accession no. NP_899064), chimp XLS (accession no. XP_009456033), mouse XLS (accession no. NP_705785), rat XLS (accession no. XP_006233650), zebrafish XLS (accession no. NP_001124069) and *Xenopus* XLS (accession no. XP_004917607) were used for alignment.

### siRNA-mediated knockdown experiments

Cells were transfected with 20 nM ON-TARGETplus siRNA SMARTpools (GE Healthcare, Little Chalfont, UK) using Lipofectamine RNAiMAX (Life Technologies) according to the manufacturer's protocol.

### Clonogenic survival assays

Seventy-two  hours following siRNA transfection, cells were replated in triplicate at low density in six-well plates. Once attached, cells were exposed to X-rays and grown for 11 days to form colonies. Colonies were fixed in 75% methanol : 25% acetic acid, before staining with 0.05% (w/v) crystal violet. Plates were scanned using an Odyssey CLX imaging system (LI-COR Biosciences, Lincoln, NE, USA) and plate intensities (as a measure of colony formation) were determined with the ColonyArea Plugin^30^ and Image-J software (National Institutes of Health, Bethesda, MD, USA). The survival fraction was determined from the plating efficiency of the treatment relative to the plating efficiency of the 0-Gy controls.

### DSB repair foci counting

Cells were transfected with siRNA reagents and then 48 h later plated at the density of 35 × 10^3^ cells per well on Lab-Tek II 8-well chamber slides (Nunc, ThermoFisherScientific, Rugby, UK) in 500 *μ*l of complete medium. After overnight incubation, cells were exposed to 1 Gy of X-rays (R320 Cabinet, X-strahl) and further cultured at 37 °C. At indicated time points cells were fixed with 4% paraformaldehyde directly on slides for 10 min at room temperature. Cells were subsequently washed with PBS and incubated at 4 °C in blocking buffer (5% normal goat serum, 0.3% Triton X-100 in PBS) for at least 30 min. Cells were further incubated with primary antibodies diluted in blocking buffer at 4 °C (rabbit anti-phospho-Ser139-H2AX Ab; Cell Signalling Technology). After overnight incubation, cells were washed in PBS at room temperature and then secondary antibodies (goat anti-rabbit Cy3-coupled Abs; Jackson ImmunoResearch, West Grove, PA, USA) were applied at room temperature in blocking buffer containing DAPI (Sigma-Aldrich) for 1 h. Finally, cells were washed in PBS and mounted in Vectashield-mounting medium (Vector Labs, Peterborough, UK). Images of nuclei were acquired on Zeiss LSM510 (Carl Zeiss Ltd., Cambridge, UK) and then individual DSB foci were counted per nucleus. To quantify DSB repair defect, 50 nuclei were scored per data point and mean values±S.D. were plotted. Data were statistically analysed using two-sided paired *T*-test.

### qPCR

Seventy-two hours post-siRNA transfection, RNA was extracted using QIAshredder columns and the RNeasy mini kits (Qiagen, Venlo, Holland) according to manufacturer's instructions. cDNA was reverse transcribed using anchored oligo dT^20^ primer and Superscript II (Life Technologies) following the manufacturer's instructions. qPCR was performed using SYBR green (Applied Biosystems, Paisley, UK) and measured on a QuantStudio 6 Flex Real-Time machine (Life Technologies). Samples were normalised to *GAPDH* mRNA and the fold change between the gene-specific siRNA and the control siRNA samples was determined.^20^ qPCR primers: *XLS* forward 5′-GAGAGTCGCTCATCAACCCC-3′, *XLS* reverse 5′-AAAGACTGCCTCTCCCCTCA-3′ *XRCC4* and *GAPDH* primer sequences were obtained from literature.^31, 32^

### Extraction of histones for Immunoblotting

Cells were resuspended in Triton extraction buffer (TEB; PBS, 0.5%(v/v) Triton X-100, 2 mM PMSF, 10 mM NaF, 1 mM Na_3_VO_4_ and 0.02% NaN_3_ , and lysed on ice for 10 min with intermittent mixing. After centrifugation at 6500 × *g* for 10 min at 4 °C, nuclear pellets were washed with half the volume of TEB and re-centrifuged. Pellets were resuspended in 0.2 N HCl and incubated overnight at 4 °C. Following centrifugation at 6500 × *g* for 10 min at 4 °C, the protein concentration of supernatants was quantified by Bradford assay (Bio-Rad).

## Abbreviations

NHEJ: non-homologous end joining
DNA-PKcs: DNA-dependent protein kinase catalytic subunit
XLF: XRCC4-like factor
XLS: XRCC4-like small protein
DSB: double-stranded DNA breaks
HR: homologous recombination
PMSF: phenylmethanesulphonyl fluoride
EtBr: ethidium bromide
IP: immunoprecipitation
